# Intake Levels of Fish in the UK Paediatric Population

**DOI:** 10.3390/nu9040392

**Published:** 2017-04-16

**Authors:** Sibylle Kranz, Nicholas R. V. Jones, Pablo Monsivais

**Affiliations:** 1 Centre for Exercise, Nutrition and Health Sciences, University of Bristol, Bristol BS8 1TH, UK; 2 UKCRC Centre for Diet and Activity Research (CEDAR), Cambridge CB2 0QQ, UK; nrvj2@cantab.net (N.R.V.J.); pm491@medschl.cam.ac.uk (P.M.)

**Keywords:** fish intake, diet quality, nutrition monitoring, NDNS-RP, child nutrition

## Abstract

The United Kingdom (UK) is an island and its culture, including diet, is heavily influenced by the maritime resources. Dietary guidance in the UK recommends intake of fish, which provides important nutrients, such as long-chain omega-3 polyunsaturated fatty acids (*n*-3 PUFA). This study was designed to describe the fish intake habits of UK children using a nationally representative sample. Dietary and socio-demographic data of children 2–18 (*N* = 2096) in the National Diet and Nutrition Survey Rolling Program (NDNS) Years 1–4 (2008–2012) were extracted. Average nutrient and food intakes were estimated. Logistic regression models were used to predict the meeting of fish intake recommendations, controlling for age, sex, income, total energy intake, and survey year. All analyses were conducted using survey routines and dietary survey weights. In this nationally representative study, 4.7% of children met the fish and 4.5% the oily fish intake recommendations; only 1.3% of the population met both recommendations. Fish intake levels did not significantly change with children’s increasing age. Higher vegetable but lower meat consumption predicted meeting the fish intake recommendations, indicating that children eating fish have better diet quality than non-consumers. Further research is needed to explore how intake behaviours can be changed to improve children’s diet quality.

## 1. Introduction

Fish contains very specific nutrients, including eicosapentanoic acid (EPA) and docosahexaenoic acid (DHA), which are considered critical, as these long-chain n(omega)-3 polyunsaturated fatty acids (PUFA) have important roles in health [1], such as heart health [2], brain [3], and eye development [4,5,6]. Since EPA and DHA are not easily synthesized in the human body and the conversion rate from medium-chain *n*-3 fatty acids, such as alpha linoleic acid (ALA), is only approximately 1% [7], dietary intake of fish is considered part of a healthy diet [8,9,10]. 

Public health guidance recommends the consumption of fish, especially oily fish (i.e., salmon, herring, anchovy, smelt, and mackerel). The *Dietary Guidelines for Americans* [11] and United Kingdom (UK) Scientific Advisory Committee on Nutrition (SACN) [12] both suggest the consumption of two servings of fish (140 g each) per week, one serving of which should be from oily fish. This is equivalent to a calculated daily average minimum of 40 g of fish, of which 20 g should be from oily fish. Meeting these intake recommendations would contribute approximately 450 mg of EPA/DHA, which is considered as the adequate intake level by the two agencies. The Joint Food and Agriculture Organization (FAO) and World Health Organization (WHO) Expert Consultation on Fats and Fatty Acids in Human Nutrition provide a similar recommendation, namely 300 mg/day EPA and DHA, of which at least 200 mg/day should be DHA [13].

Studies of children’s diets in Germany indicated that children consuming even small amounts of fish had significantly improved long-chain *n*-3 PUFA consumption levels compared to non-consumers [14]. A study of national fish consumption trends in American children reported overall low intake of fish; moreover, only one of the three most frequently consumed fish species was an oily variety [15]. Likewise, another analysis of the diets of one through five year old American children found very low intake of fish and long-chain *n*-3 PUFA [16].

The countries of the UK (England, Wales, Scotland, and Northern Ireland) are geographically maritime—islands surrounded by waters that are traditionally harvested for fish and seafood. Although slightly varied between these four countries, the historic diet culture in the UK includes many fish- and seafood-based dishes, such as baked or pickled fish, seafood salads or pies, and smoked/dried fish. The most recognized dishes internationally include “fish and chips” (battered and deep fried cod, haddock or plaice served with large-cut potato fries), “kippers” (salted and smoked herring), or smoked mackerel. These dishes are found on the menu of most eateries in the UK. They are offered as low-cost street-food but also in higher-end restaurants as “traditional fare”.

The national dietary intake guidance for the UK is issued by the Food Standards Agency in form of the “The Eatwell Plate” and reflects the SACN recommendations for fish and oily fish [17]. Survey data shows that fish intake is recognized as a healthy choice by young people, although opinions somewhat vary [18]. Despite the relatively high availability of fish and seafood and consistent public health guidance to consume more fish, just like American children, UK children may not meet the intake recommendation. 

The primary goal of this study was to use nationally representative data to examine the fish and seafood consumption of children in the UK, and to compare reported consumption levels with the intake recommendations for total fish and oily fish. A secondary goal was to examine the association of fish intake to other indicators of diet quality, in this case vegetable and meat intake.

## 2. Materials and Methods

### 2.1. Population Sample

Data from 2–18 year-old children (*N* = 2096) of the survey years 1–4 of the National Diet and Nutrition Survey rolling programme (NDNS-RP, 2008–2012) were included in this study. Children’s age was used to stratify the population in three distinct age groups: 2–5 year olds, 6–11 year olds, and 12–18 year olds. All analyses were conducted for the total sample, as well as separately for those who reported consuming any fish. NDNS is publicly available and de-identified data, thus this study does not fall under the scope of research in human subjects. 

Dietary, demographic, and socioeconomic data were obtained from the dataset. Demographic and socio-economic data (sex, race/ethnicity, and income) were used to describe the sample. Income was not available for 21 (1%) children in the sample, leaving *N* = 2075 children for whom the household income was categorized and equalised using a method previously used [16]. Income was coded as missing when household income was not provided.

### 2.2. Nutritional Variables

To estimate dietary intake, four consecutive 24-h estimated diet diaries (records) were collected from participants. Participants kept dietary intake records for four consecutive days. Dietary intake data collection was conducted year-round. No information as to the season of the data collected here is available. Weekend days were over-sampled in year one of the survey and under-sampled in the remaining years of the dataset used here, thus providing a fair estimate of usual daily intake of the participants. Each participant aged 12 and older was instructed on how to complete his or her own diary using household measures (i.e., measuring cups and spoons) and information from product packages to estimate the amounts of food and drink consumed. Children 16 and older were asked to estimate the portions of food and drink consumed by referring to a photo booklet of commonly-consumed items. For children younger than 12, parents or care-givers were asked to complete the diary, with information from the child. By the second or third diary day, survey staff met with the participant to review the diary days completed so far and provide feedback for improving the quality of diet data collection for the remaining diary days. Daily average food group and nutrient consumptions are reported in the dataset. Completed diaries were coded and entered by trained survey staff. Data were entered into the MRC-Human Nutrition Research dietary assessment system DINO (Diet IN Nutrients Out), an analysis system written in Microsoft Access. Nutrient intake assessed by DINO is based on a food composition database derived from UK food tables and supplemented with additional information from the food industry. Details of the use of DINO to estimate nutrient intakes in NDNS has been reported [19]. 

For the purpose of this study, total energy (kilocalories per day (kcal)), food group (in percent of total energy consumed for protein, fat, carbohydrates, and total sugars) and individual food items (in grams per 1000 kcal consumed or fruits and vegetables, total meat, red meat, white meat, eggs, nuts and seeds, total fish, white fish, oily fish, canned tuna, and shellfish) were calculated. This approach allows for comparison across age groups, which consume different levels of total energy and therefore increasing amounts of each food group with increasing age. Based on the reported diet, children were classified as “fish consumers” if they consumed any amount of fish or seafood (NDNS Variable 33 “White fish coated or fried”, Variable 34 “Other white fish, shellfish and fish dishes”, and Variable 35 “Oily fish”) or as non-consumers otherwise. All data were reported for all children and disaggregated by age group for the total sample and the fish consumers and non-consumers separately. In the NDNS dataset, the *n*-3 PUFA are estimated as one variable, thus, no differentiation between the intake of long-chain *n*-3 PUFA, such as EPA and DHA, and short- or medium-chain PUFA can be made.

### 2.3. Statistical Analysis

All analyses were conducted using survey routines and the dietary survey weights to maintain the nationally representative character of the data. Analyses were conducted in STATA Version 13 (STATA Corporation, College Station, TX, USA). Mean consumption of food groups and nutrients was calculated for the total population and for each age group separately and reported as mean ± standard error. Intakes of macronutrients were expressed as percent energy and food groups were expressed on a gram (g) per-1000 kcal basis in order to facilitate comparisons across age groups. Since fish and seafood consumption was highly skewed, so median and interquartile range were estimated to describe the consumption in the population. Non-parametric test for trend across ordered groups was used to determine if consumption increased or decreased with increasing age across three age groups (2–5, 6–11, 12–18 years old). Significant differences in mean consumption between the group of children who consume fish and non-consumers was determined using the *lincom* command, the survey routine equivalent to the Student’s *T*-test to compare means. Logistic regression modelling was employed to determine the contributors to children’s odds of (a) meeting the dietary intake recommendation for total fish (40 g/day); (b) for oily fish (20 g/day); and (c) for any consumption of fish. All models controlled for age, sex, household income, total energy intake, and NDNS survey year. Statistical significance was assumed using a 95% level of significance.

## 3. Results

Based on the nature of this study, the demographic profile of the population included in the sample is representative of all children in the UK (data not shown). More than half of the population (55%, *N* = 1142) consumed any fish/seafood during the four days of dietary intake measurement (Table 1). Of those, 4.7% met the total fish intake recommendation, 4.5% the oily fish intake recommendation, and only 1.3% (*N* = 28 children) of 2–18 year olds met both (the total fish and the oily fish intake recommendations). 

Fish and seafood intake was highly skewed, hence, both mean and standard error (SE) as well as median and interquartile range (IQR), the 25th and 75th percentile of intake, were calculated. Total fish and seafood consumption was low (mean ± SE = 10.41 ± 0.42; median and IQR: 3.78 g/day and 0; 16.5 g/day); oily fish consumption was even lower (mean ± SE = 1.78 ± 0.17; median and IQR: 0 g/day and 0; 0 g/day); the 90th percentile of oily fish intake was only 2.55 g/day.

Analysis of the average dietary intake of foods and food groups in the total sample and the subsamples of fish consumers, and those children meeting the intake recommendations, showed that children who consumed fish had better diet quality (Table 2). Those children consumed more vegetables and less meat. Most nutrient and food group intake vary with age in the total population, in that total energy, meat density (red and white meat density) and shellfish increased while percent of energy from added sugar, fruit and vegetable density, total- and white fish density decreased. Comparison of intake between fish consumers and non-consumers showed that children who consume fish have higher fruit and vegetable density in their diet and lower meat density.

Logistic regression models showed that neither household income nor ethnic group was associated with total fish or oily fish intake (data not shown) and subsequent modelling including age, sex, equalised household income, total energy consumed and NDNS survey year as covariates (Table 3). 

Consuming any fish was strongly and positively associated with being in the medium or highest tertile of vegetable intake and negatively associated with eating the medium or highest tertile of meat (OR = 1.55, 95% CI 1.19–2.12; OR = 1.88, 95% CI 1.39–2.58; OR = 0.72, 95% CI 0.55–0.98; OR = 0.47, 95% CI 0.34–0.66, respectively). Consuming the recommended amount of two servings of fish was significantly and positively predicted by consuming the highest tertile of vegetables (OR = 2.51, 95% CI 1.30–4.84) and significantly but negatively associated with consuming the highest tertile of meat (OR = 0.32, 95% CI 0.16–0.64). The odds of meeting the intake recommendation for oily fish was increased with eating the highest tertile of vegetables (OR = 3.49, 95% CI 1.21–9.96) and the medium or highest tertile of meat consumption (OR = 2.84, 95% CI 1.20-6.75 and OR = 2.65, 95% CI 1.90–7.84, respectively).

## 4. Discussion

Recommendations for fish intake, especially those focussing on oily fish, are in part designed to promote adequate intakes of EPA and DHA in children and adults. Low intake of EPA/DHA is commonly observed in the Western diet [17] and might negatively affect children’s brain development and function [18,19]. Data from observational studies indicated that low levels of EPA/DHA may be associated with rising prevalence of childhood developmental disorders, such as attention-deficit hyperactivity disorder and dyslexia [20]. Overall, EPA and DHA have a recognized role in health and disease prevention [1,2,3,4,5,6]. As our results show, the proportion of children in the UK meeting the intake recommendations for total fish and oily fish intake is extremely small. This is reason for concern.

Although small amounts of EPA and DHA can be found in foods other than fish, those foods (predominantly eggs and dark poultry meats) are also very high in saturated fatty acids [21]. Although we showed that a very large proportion of 2–18 year-old children in the UK fail to meet the intake recommendation for total fish and/or oily fish, we are not able to directly assess if children meet the targeted daily minimum consumption amounts for EPA and DHA because EPA and DHA are not reported separately from medium- and short-chain fatty acids in the NDNS data set. This particular problem with the UK national dietary data survey system has been noted previously [22]. It would be very beneficial for policy purposes if future releases of the NDNS nutrient data wold be modified to allow a direct measurement of the individual dietary fatty acids. However, it is noteworthy to point out that the measurement of EPA and DHA in free-living individuals is a challenge, due to tissue storage, conversion rate, and saturable plasma levels [20]. In the future, other approaches might be possible to better estimate fish intake, such as a metabolomic study of biomarkers of meat and fish intake [21].

Despite the lack of direct measurement of EPA and DHA intake levels in this sample, our analysis showed that the vast majority of 2–18 year old children in the UK fail to meet the intake recommendations for fish. Interestingly, the density of fish in the diet increased with increasing age for most categories of fish, but consumption of white fish dropped and oily fish remained almost the same in 12–18 year olds while intake of canned tuna and shellfish experienced a marked increase compared to the two younger age groups. While the similar findings in the US paediatric population [22] may be explained by the relatively low availability of affordable fish, especially oily fish, the same phenomenon in the UK must have different reasons and begs to be explored further. In the US, especially young children from low-income households may have limited access to fish, due to their mothers dietary intake practices, which have been shown to be low in fish and other healthy items [23]. However, as the authors for that research report explain, there is severe lack of evidence on the possible venues to rectify the problem of low diet quality in low-income women. Some possible contributing factors may be the lack of access (i.e. family ability to obtain seafood from local vendors) and availability (i.e. the quantity and quality of food provided), the relatively higher cost of some oily fish compared to (for example) cheaper processed meats [23]. Also, parents’ and carers’ knowledge and ability to prepare fish, as well as their role-modelling of eating fish and seafood [24] may contribute. Other changes in local culture, such as the lack of family meals and a preference to consume foods from other cuisines are potential contributing to the low intake of fish in children. These phenomena may also be the underlying cause of reduction of fish intake in Mediterranean countries, which used to be traditionally high in fish intake [24,25]. A published research report for an intervention study to increase dietary EPA and DHA consumption of preschool-age children showed that the substitution of meats usually used for the lunch meal in child care settings can be replaced with oily fish [25]. Since oily fish, such as salmon, herring, and sardines, are so high in EPA and DHA that even a small increase in intake results in significantly improved intakes, adding fish to mixed dishes, such as pasta and sauce, may significantly improve children’s EPA and DHA consumption. 

One possible limitation of this study was the use of four estimated dietary records to estimate intake. Although this method has been accepted for large nutrition surveys, intake of rarely consumed foods, such as seafood, may be misrepresented using this somewhat short-term data collection method. On the other hand, the level of detail provided by multiple-day records is superior to diet information obtained from long-term food frequency questionnaires. Compared to the intake estimates used in US national surveys (using two 24-hour recalls), the use of four days of food records doubled the odds of capturing those rarely consumed foods. Ideally, future research on fish intake would include a biomarker of intake levels, such as trimethylamine-N-oxide [21].

One major strength of this study was the use of a nationally-representative data set, which allows generalizability of the results. Some might expect that younger children have different intake patterns than teenagers. However, the consumption levels of all fish and seafood types available in the dataset show that intake increases with age, with the exception of white fish. 

A key finding was that only approximately 2% of the population met the recommendations for both total fish and oily fish intake. Interestingly, our data show that children who consumed any fish or seafood also had healthier eating patterns, characterized by higher vegetable intake but lower meat intake. This relationship between higher vegetable but lower meat intake showed a significant trend, in that the odds for meeting the fish intake recommendations were even better in the children consuming in the highest tertile of vegetable compared to the medium tertile. Likewise, the odds for meeting the intake recommendations were lower for the children consuming in the highest tertile for meat intake compared to the medium tertile of meat consumption. This finding suggests for the first time that in the UK paediatric population, fish intake may be a useful proxy for overall healthy eating patterns.

Results of this study indicate that programmes and policies to promote the consumption of fish in the UK are not heeded. Although some might recommend more frequent and larger amounts of fish intake in the paediatric population, even a small intake of predatory fish raises the concern for the possible nutrition-toxicity issues [26]. Supplying sufficient amounts of safe fish to meet EPA and DHA intake recommendations may also be at odds with the sustainability of marine ecosystems [27]. At the same time, exploring other dietary sources of EPA and DHA that are low-cost, widely available, and acceptable to children might be beneficial for all children, not only in the UK. 

## 5. Conclusions

Our study indicates that children in the UK do not consume sufficient amounts of fish. However, due to the lack of data on the EPA and DHA consumption, it is not clear whether or not the low fish intake is associated with inadequate levels of these two important nutrients for brain development and functioning. Further research is needed to pursue this question and to identify the barriers to fish intake in a population of traditionally high fish- and seafood diets.

## Figures and Tables

**Table 1:** Proportion of children meeting the recommended fish and seafood intake of the “Eatwell Plate” UK national intake recommendations in the National Diet and Nutrition Survey 2008–2012 in the total sample (*N* = 2075).

Characteristic	Total Sample (*N* = 2096)	2–5 Year Olds (*N* = 634)	6–11 Year Olds (*N* = 664)	12–18 Year Olds (*N* = 798)
Fish consumers ^1^	54.9	62.9	58.3	45.5
Meet fish recommendation (≥40 g of fish/day)	4.2	2.5	4.6	5.3
Meet oily fish recommendation (≥20 g of fish/day)	4.0	2.4	4.8	4.7
Meet fish and oily fish recommendations	1.3	0.6	1.4	1.0

^1^ Includes NDNS food groups “White fish coated or fried”, “Other white fish, shellfish and fish dishes”, and “Oily fish”.

**Table 2:** Estimated intakes of select food groups and foods^†^ by children in the National Diet and Nutrition Survey 2008–2012 in the total sample and by age group (*N* = 2096).

	All Children	2–5 Year Olds	6–11 Year Olds	12–18 Year Olds	Test for Trend ^‡^ *p*-Value
Total sample (*N* = 2096)					
Total energy (kcal/day)	1554 ± 14	1205 ± 17	1608 ± 18	1801 ± 24	<0.001 ***
Protein (% energy/day)	14.8 ± 0.8	15.1 ± 0.1	14.4 ± 0.1	14.8 ± 0.1	0.172
Fat (% energy/day)	33.7 ± 0.1	33.8 ± 0.3	33.4 ± 0.2	33.8 ± 0.2	0.783
Carbohydrates (% energy/day)	51.2 ± 0.2	51.2 ± 0.3	52.2 ± 0.2	50.5 ± 0.3	0.011 *
Energy-adjusted intakes of food groups (g/1000 kcal consumed)					
Fruits and vegetables	126.3 ± 2.4	158.9 ± 5.1	129.4 ± 3.7	96.8 ± 2.7	<0.001 ***
Total meat	47.2 ± 0.8	37.7 ± 4.1	46.4 ± 1.1	44.8 ± 1.4	<0.001 ***
Red meat	30.6 ± 0.7	25.7 ± 1.2	30.6 ± 1.1	34.8 ± 1.2	<0.001 ***
White meat	16.6 ± 0.5	12.1 ± 0.7	15.8 ± 0.7	21.0 ± 0.8	<0.001 ***
Eggs	6.9 ± 0.4	6.8 ± 0.6	6.1 ± 0.6	7.6 ± 0.7	0.345
Nuts and seeds	0.6 ± 0.1	0.7 ± 0.6	0.6 ± 0.1	0.6 ± 0.1	0.263
Total fish	7.1 ± 0.3	7.9 ± 0.5	7.2 ± 0.5	6.2 ± 0.4	<0.001 ***
White fish	4.0 ± 0.2	4.9 ± 0.4	4.2 ± 0.4	2.3 ± 0.2	<0.001 ***
Oily fish	1.2 ± 0.1	1.1 ± 0.2	1.3 ± 0.2	1.1 ± 0.2	0.164
Canned tuna	1.6 ± 0.1	1.6 ± 0.3	1.1 ± 0.1	1.9 ± 0.3	0.152
Shellfish	0.6 ± 0.1	0.2 ± 0.1	0.6 ± 0.1	0.8 ± 0.2	<0.001 ***
Consumers of fish/seafood (*N* = 1142)					
Total energy (kcal/day)	1522 ± 18 ⊥	1211 ± 18	1621 ± 26	1776 ± 34	<0.001 ***
Protein (% energy/day)	15.1 ± 0.1 ⊥	15.3 ± 0.2 ⊥	14.7 ± 0.2 ⊥	15.2 ± 0.2 ⊥	0.812
Fat (% energy/day)	33.8 ± 0.2	33.7 ± 0.4	33.6 ± 0.3	34.2 ± 0.3	0.875
Carbohydrates (% energy/day)	50.9 ± 0.2 ⊥	51.0 ± 0.4	51.8 ± 0.3 ⊥	50.0 ± 0.4	0.055
Energy-adjusted intakes of food groups (g/1000 kcal consumed)					
Fruits and vegetables	135.3 ± 3.2 ⊥	162.9 ± 0.006.2	138.3 ± 4.1 ⊥	100.4 ± 3.7	<0.001 ***
Total meat	43.4 ± 1.1 ⊥	35.3 ± 1.8 ⊥	43.7 ± 1.5 ⊥	52.3 ± 2.0 ⊥	<0.001 ***
Red meat	27.6 ± 0.7 ⊥	23.6 ± 1.4 ⊥	28.4 ± 1.3 ⊥	31.2 ± 1.6 ⊥	<0.001 ***
White meat	15.8 ± 0.6	11.7 ± 1.0	15.3 ± 0.8	21.1 ± 1.3	<0.001 ***
Eggs	6.6 ± 0.5	7.0 ± 0.8	6.1 ± 0.9	6.9 ± 0.8	0.247
Nuts and seeds	0.7 ± 0.1 ⊥	0.7 ± 0.2	0.7 ± 0.2 ⊥	0.6 ± 0.2	0.153
Total fish	12.9 ± 0.4	12.6 ± 0.8	12.5 ± 0.6	13.7 ± 0.7	0.064
White fish	4.0 ± 0.2	7.8 ± 0.6	7.2 ± 0.5	5.0 ± 0.5	<0.001 ***
Oily fish	1.2 ± 0.1	1.8 ± 0.3	2.3 ± 0.3	2.5 ± 0.4	0.406
Canned tuna	1.6 ± 0.1	2.6 ± 0.5	1.9 ± 0.2	4.4 ± 0.5	<0.001 ***
Shellfish	0.6 ± 0.1	0.3 ± 0.1	1.1 ± 0.2	1.9 ± 0.4	<0.001 ***
Non-consumers of fish/seafood (*N* = 954)					
Total energy (kcal/day)	1594 ± 22 ⊥	1196 ± 30	1590 ± 25	1821 ± 34	<0.001 ***
Protein (% energy/day)	14.4 ± 0.12 ⊥	14.7 ± 0.2 ⊥	14.1 ± 0.2 ⊥	14.5 ± 0.2	0.314
Fat (% energy/day)	33.6 ± 0.2	34.0 ± 0.5	33.2 ± 0.4	33.6 ± 0.4	0.565
Carbohydrates (% energy/day)	51.5 ± 0.2 ⊥	51.4 ± 0.5	52.8 ± 0.4	50.9 ± 0.4	0.062
Energy-adjusted intakes of food groups (g/1000 kcal consumed)					
Fruits and vegetables	115.5 ± 3.7 ⊥	152.2 ± 8.7	116.9 ± 6.8 ⊥	93.8 ± 3.9	<0.001 ***
Total meat	51.9 ± 1.2 ⊥	41.8 ± 2.2 ⊥	50.1 ± 1.9 ⊥	58.8 ± 1.8 ⊥	<0.001 ***
Red meat	34.4 ± 1.1 ⊥	29.2 ± 1.9 ⊥	33.6 ± 1.8 ⊥	37.8 ± 1.7 ⊥	0.002 **
White meat	17.6 ± 0.7	12.6 ± 1.1	16.5 ± 1.1	21.0 ± 1.1	<0.001 ***
Eggs	7.1 ± 0.6	6.6 ± 1.0	6.0 ± 0.8	8.1 ± 1.0	0.945
Nuts and seeds	0.5 ± 0.1 ⊥	0.6 ± 0.2	0.3 ± 0.1 ⊥	0.6 ± 0.1	0.878

^†^ Includes NDNS food groups “White fish coated or fried”, “Other white fish, shellfish and fish dishes”, and “Oily fish”; ^‡^ across ordered groups by age group; Asterisks indicate significant trend with increasing age * *p* < 0.05; ** *p* < 0.01; *** *p* < 0.0001; ⊥ significantly different between fish consumers and non-consumers.

**Table 3:** Association (odds ratios and 95% confidence intervals) of children’s dietary characteristics for children, who (a) are fish consumers and (b) meet the fish intake recommendation of 280 g/week (or 40 g/day), and (c) meet the oily fish intake recommendation of 140 g/week (or 20 g/day) (*N* = 2096).

		Odds Ratio	95% Confidence Interval
(a) Consume any fish/seafood			
Tertile of vegetable	Lowest	1.00	
	Medium	1.55 **	1.19, 2.12
	Highest	1.88 ***	1.39, 2.58
Tertile of meat	Lowest	1.00	
	Medium	0.72 *	0.55, 0.98
	Highest	0.47 ***	0.34, 0.66
(b) Meet fish intake recommendation			
Tertile of vegetable	Lowest	1.00	
	Medium	0.99	0.45, 2.16
	Highest	2.51 **	1.30, 4.84
Tertile of meat	Lowest	1.00	
	Medium	0.55	0.27, 1.09
	Highest	0.32 **	0.16, 0.64
(c) Meet oily fish intake recommendation			
Total fish intake (g/day)		1.07 ***	1.05, 1.09
Tertile of vegetable	Lowest	1.00	
	Medium	1.89	0.67, 5.97
	Highest	3.47 **	1.21, 9.96
Tertile of meat	Lowest	1.00	
	Medium	2.84 **	1.20, 6.75
	Highest	2.65 *	1.90, 7.84

Values were significantly different at: * *p* < 0.05; ** *p* < 0.01; *** *p* < 0.0001, all models controlling for age, sex, total energy intake, equalized household income, and NDNS survey year.
